# A Low-Cost Environmental Monitoring System: How to Prevent Systematic Errors in the Design Phase through the Combined Use of Additive Manufacturing and Thermographic Techniques

**DOI:** 10.3390/s17040828

**Published:** 2017-04-11

**Authors:** Francesco Salamone, Ludovico Danza, Italo Meroni, Maria Cristina Pollastro

**Affiliations:** ITC-CNR, Construction Technologies Institute, National Research Council of Italy, Via Lombardia, 49, 20098 San Giuliano Milanese (MI), Italy; ludovico.danza@itc.cnr.it (L.D.); italo.meroni@itc.cnr.it (I.M.); pollastro@itc.cnr.it (M.C.P.)

**Keywords:** indoor environmental quality, indoor air quality, indoor thermal comfort quality, internet of things, DIY, IoT, nearable, environmental monitoring system, thermography, additive manufacturing

## Abstract

nEMoS (nano Environmental Monitoring System) is a 3D-printed device built following the Do-It-Yourself (DIY) approach. It can be connected to the web and it can be used to assess indoor environmental quality (IEQ). It is built using some low-cost sensors connected to an Arduino microcontroller board. The device is assembled in a small-sized case and both thermohygrometric sensors used to measure the air temperature and relative humidity, and the globe thermometer used to measure the radiant temperature, can be subject to thermal effects due to overheating of some nearby components. A thermographic analysis was made to rule out this possibility. The paper shows how the pervasive technique of additive manufacturing can be combined with the more traditional thermographic techniques to redesign the case and to verify the accuracy of the optimized system in order to prevent instrumental systematic errors in terms of the difference between experimental and actual values of the above-mentioned environmental parameters.

## 1. Introduction

The relation between indoor environmental quality (IEQ) and building energy simulation (BES) [1] is a topic of active investigation [2,3]. A significant effort has been spent to identify different methods of calculation [4], semantic data models [5,6] and systems of measurement that are more and more advanced and integrated using non-invasive technologies in order to maximize the well-being of occupants and to reduce the associated energy consumption [7].

This article illustrates the additive manufacturing and thermography techniques used to evaluate the thermal irregularities and to optimize the functional distribution of a series of sensors placed within an integrated device, nEMoS (nano Environmental Monitoring System), for monitoring indoor environmental variables [8].

Local variations in temperature, in fact, may lead to an alteration of the measurement of heat-sensitive sensors and, in extreme cases, to a malfunction or breakage of the sensors. In both cases, it is useful to proceed to an analysis of the temperature distribution in order to optimize the component layout [9].

There are three different typologies of temperature measurement techniques: invasive, semi-invasive, and non-invasive [10]. The last one may be carried out by exploiting the physical principle whereby anybody, and everything, at a temperature above absolute zero transmits infrared radiation corresponding to the temperature of the object. Such a temperature depends on molecular motion: the greater the intensity of the movement, the greater the temperature of the object. Through thermography it is possible to measure the infrared radiation emitted by an object and its temperature distribution.

Infrared thermography (IRT) is applied in all fields where it is important to know the surface temperature of a body or parts of it [11]. Through appropriate correlations it is possible to determine the effect associated with a particular temperature. In particular, IRT is applied in the building sector [12], for example, to perform non-destructive assessment to quantify energy losses due to thermal bridging [13] or to characterize the dynamic behavior of a wall [14]. Other application of IRT include the possibility to detect air leakage through the building envelope in place of the classical blower door test [15]. Another application in the human thermoregulation field includes the possibility to use IRT to monitor the skin temperature of the face and then to assess the related comfort sensation [16].

The development of new technological solutions and the use of pervasive technologies, such as 3D printing, has allowed the proliferation of smart instruments that have been applied in different contexts, even for research purposes, with satisfactory results. This logic is the basis for the development of an open source colorimeter [17], an open source control system for a solar plant [18], and a new and inexpensive pyranometer [19]. These are only some example of research instruments developed following the DIY approach that have been shared, in line with the maker movement philosophy. The use of low-cost sensors require a verification phase to understand the behavior of the instrument used. In fact the low-cost sensors often have incomplete technical specifications which, as can be read below, may negatively affect the outcome of hardware development. Researchers, makers, and followers of the DIY approach should always keep in mind this aspect. They may consider this paper as a possible reference when they want to develop something new.

The sharing of information, an established practice in the maker movement [20,21,22], has allowed people with different backgrounds to approach electronics, applying the Do-It-Yourself [23,24] attitude to develop specific solutions. The developed devices are often called "smart” objects because they allow active interaction with the environment [25,26] because they are equipped with modules and they adopt specific communication protocols [27,28]. These smart devices are, substantially, the nodes of a network that is known as the Internet of Things [29,30,31], often expressed with the acronym IoT. Many of these objects are used in the domestic environment, such as smart TVs, smart thermostats, and smart surveillance systems.

The DIY and IoT approaches have been applied to the design and construct of nEMoS nearable device for the monitoring of the IEQ. The term “nearable” (or nearable technology), used for the first time in 2014 as part of a marketing campaign, is now used to uniquely identify the idea of smart objects that can be equipped with a variety of sensors and can work as transmitters to broadcast digital data. The IEQ is a holistic concept including the indoor air quality (IAQ), the indoor lighting quality (ILQ), and indoor acoustic comfort [32,33], besides the indoor climate quality (ICQ). Today, different devices are used to assess the IEQ. ITC-CNR has developed an integrated tool, called nEMoS [34], for assessing the IEQ based on the cost-effectiveness and reliability of the monitored data because it uses only a microcontroller and low-cost sensors that have been compared with professional sensors [34]. The main difference between the low-cost sensors and professional sensors is related to the calibration certificate which is provided only for the last ones. Moreover, the professional sensors have more exhaustive and complete technical specifications than those of low-cost sensors. In the previous paper [34], all sensors used are compared with the equivalent professional sensors but no descriptions are reported on how and why the case of the 3D printed device was developed with this form. This article focuses on the steps that have led to the optimization of the case of this integrated device through the combined use of the new pervasive technologies of 3D printing (typically used in the DIY world) and more “classical” thermographic techniques (typically used by professionals). It is structured as follows: Section 2 summarizes the materials and methods; Section 3 reports the results of the first test of thermal analysis and the irregularity in the measurement of air temperature, relative humidity, and radiant temperature considering the first case; Section 4 reports the results of the calibration phase of the sensors in order to avoid measuring errors due to faults; Section 5 displays the optimized case and the results of the new tests. Finally, in Section 6, the conclusions are reported.

## 2. Materials and Methods

Table 1 summarizes the hardware of nEMoS device.

**Figure 1:**
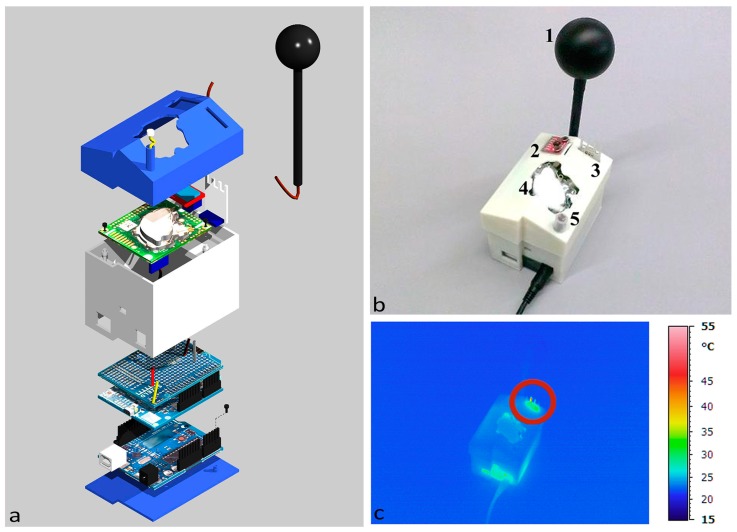
First case: (**a**) assembling scheme; (**b**) position of sensors; and (**c**) thermal imaging at starting time.

For air temperature and relative humidity sensing, two different integrated sensors were used: the HIH6130 (Honeywell, Golden Valley, MN, USA) for the 1st case and a DHT22 (Aosong (Guangzhou) Electronics Co., Ltd., Guangzhou, China) for the 2nd case. Prior to carrying out the phases of design and construction of the first prototype of the case of nEMoS device, all low-cost sensors are tested for direct comparison with professional sensors in a controlled environment [34]. The case consists of three main parts: a base, the side structure with the housings for the various sensors and a top lid. It was conceived in an extremely compact way, made with 3D printing, with dimensions (L × H × W) equal to 8.5 × 6 × 7.5 cm. The model, made in 3D CAD, was printed with successive deposits of ABS, starting from the lower layer. The dimensional tolerances were in the order of +/−0.5 mm along the axes *x*, *y*, and *z* (Figure 1a). The Dimension Elite 3D printer used is based on FDM (fused deposition modeling) technology.

Figure 1a shows the assembly of the monitoring station. All of the sensors are positioned on the top (Figure 1b): 1, the globe thermometer, 2, the air temperature and relative humidity sensor (HIH6130), 3, the anemometer (wind sensor made of a modern device), 4, the CO_2_ concentration sensor (k-30 made by CO_2_ meter,) and 5, the LDR (that works as a luxmeter).

## 3. Thermal Analysis—First Test

In order to evaluate the behavior of the device and to detect instrumental systematic errors in real working conditions, an analysis was performed with the aid of a portable infrared camera (Figure 1c), the Avio TVS-700 whose optical system is sensitive to infrared radiation with a wavelength in the range of 2–14 μm, the range in which the materials generally used in electronics radiate energy, thus showing their thermal behavior [35]. The analysis was carried out for two different cases in two distinct phases: at the beginning of the test and after one day of operation.

At the beginning of the test (Figure 1b) it is possible to notice an almost constant distribution of temperature along the case, slightly lower than 25 °C. Two localized increases in temperature can be observed at the resistor of the anemometer, as well as in the lower part near the power jack, where the voltage regulator of the Arduino UNO is located.

**Figure 2:**
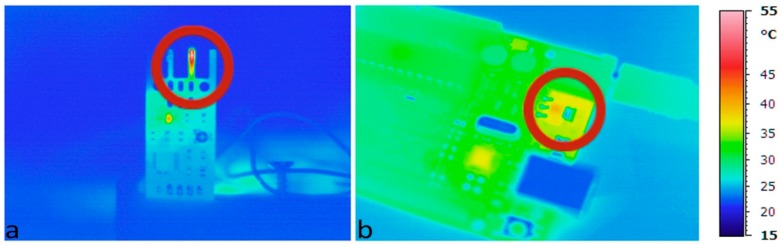
Thermal imaging: (**a**) anemometer; and (**b**) voltage regulator of the Arduino UNO.

A hot-wire anemometer is used to measure the air speeds that are normally medium-low in confined spaces. The temperature of its heated element is maintained slightly above 50 °C (Figure 2a). The air speed changes are defined measuring the current required to maintain the heated element at a constant temperature.

The system is powered by a 12 V (Vin) AC/DC adapter. The voltage regulator mounted on the Arduino UNO board provides an output voltage of 5 V (Vout). The current (I) through nEMoS is about 160 mA. The power dissipated in the voltage regulator, almost equal to 1 W, is given by the product of the voltage that passes through it, multiplied by the current through it (Equation (1)):

(1) $$P=\left(V_{in}-V_{out}\right)\times I$$

The voltage regulator mounted on the Arduino UNO board is the ncp1117st50t3g type in the 318-H casing version. The value of the junction-to-case thermal resistance (Rt) obtained from the datasheet is equal to 15 °C/W.

Considering a surrounding temperature (Tsurr) of about 24.5 °C, the temperature obtained for the case of the voltage regulator (Tc) is equal to about 40 °C (Equation (2)):

(2) $$T_{c}=T_{surr}+P\times R_{t}$$

The thermographic analysis (Figure 2b) highlights how effectively the temperature value in the proximity of the voltage regulator can be compared to the calculated value.

**Figure 3:**
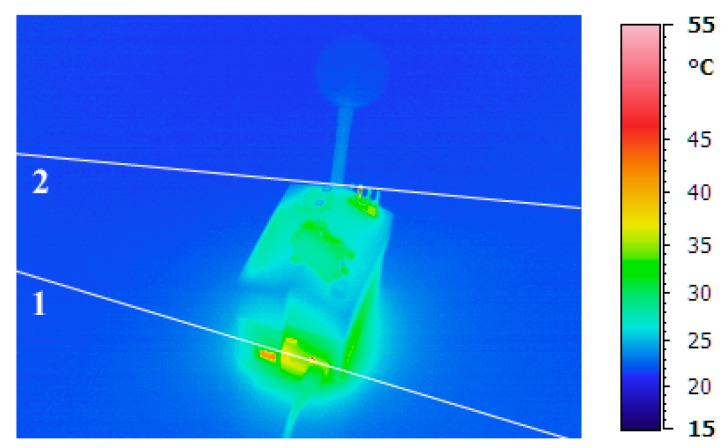
First case, thermal imaging after 24 h.

After approximately 24 h of use of the first case of nEMoS (Figure 3) a maximum temperature slightly above 50 °C can be observed on the top of the case at the anemometer level, as well as a temperature of about 40 °C in the lower part near the power jack, where the voltage regulator is located. However, the integrated temperature and humidity sensor records a temperature of about 29 °C greater than the ambient temperature.

**Figure 4:**
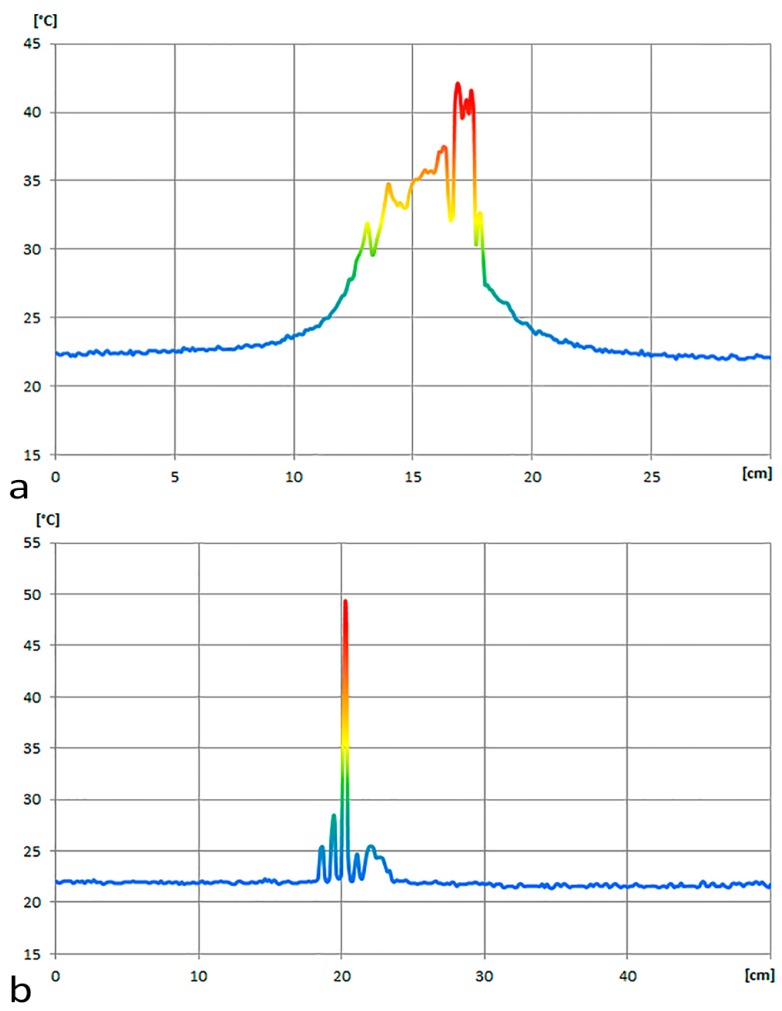
Temperature distribution: (**a**) Profile 1; and (**b**) Profile 2.

Profile 1 highlights the disturbance in temperature distribution due to the voltage regulator (Figure 4a). It can be observed how a disturbance of the temperature range due to the voltage regulator and to the nearby power jack extends to a diameter of about 10 cm. Profile 2 shows the field of perturbation due to the anemometer (Figure 4b). In this case the perturbation of the temperature due to the anemometer extends to a diameter of about 6 cm.

**Figure 5:**
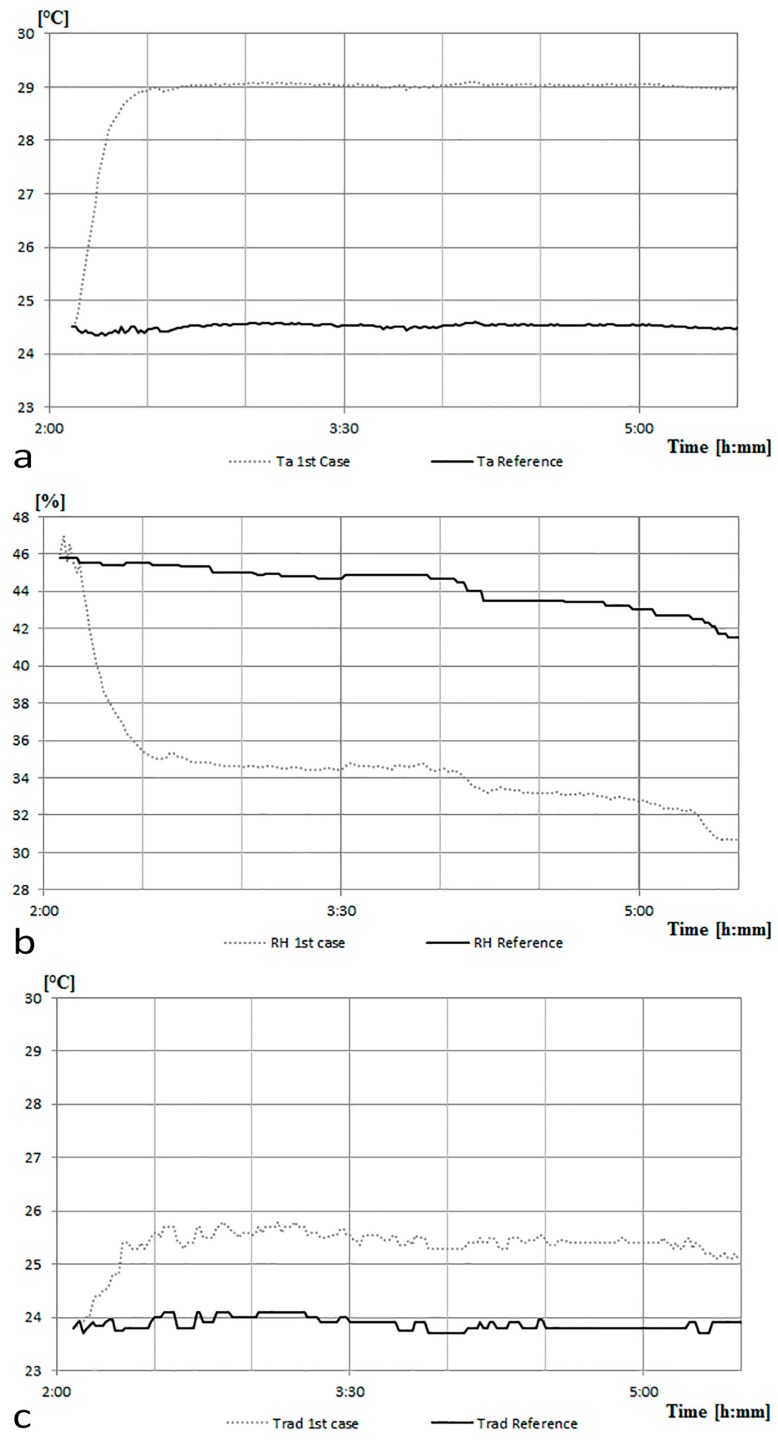
The first case vs the reference sensors: (**a**) air temperature (Ta) data; (**b**) relative humidity (RH) data; and (**c**) radiant temperature (Trad) data.

The values recorded by the device are compared (Figure 5) to those measured by two reference sensors: a thermoigrometric sensor (Ta Reference in Figure 5a; RH Reference in Figure 5b) consisting of a four-wire PT100 sensor and a thin film capacitive relative humidity sensors (accuracy: RH ± 2%, Ta < ± 0.1 °C; average response time: 8 s; resolution: RH 0.1%, Ta = 0.015 °C); a globe thermometer (Trad Reference in Figure 5c) consisting of a four-wire PT100 sensor with copper globe black color (accuracy: ±0.1 °C; average response time: <10 s; resolution: 0.01 °C).

As it may be noticed, a rapid increase in measured temperature values occurs during the first minutes elapsed after the start of the trial. The increase in temperature of the device affects the temperature measurement of the sensor. After one hour of operation the curve tends to be almost constant and equal to about 29 °C, which provides evidence of the fact that it is necessary to wait about an hour to reach thermal equilibrium. On the whole, according to the characteristics of the case described above, the system records a temperature about 4.5 °C higher than that of the surrounding air.

The aforementioned irregularity is confirmed also regarding the detection of RH and Trad. In order to avoid measuring errors due to failures of the sensors, a calibration phase was conducted. This activity is necessary to ensure that measuring errors are only due to the presence of the nearby hot wire anemometer.

## 4. Calibration Phase of the Sensors

The calibration activity was conducted to avoid the possibility of measurement errors and to assess the accuracy of the integrated Ta and RH sensors (HIH-6130 and DHT22) and the Trad sensor (LC_G).

### 4.1. Ta and RH

In the calibration phase, a DHT22 sensor was also considered as an alternative to the HIH-6130. The technical characteristics of these two integrated sensors are reported in Table 2.

**Figure 6:**
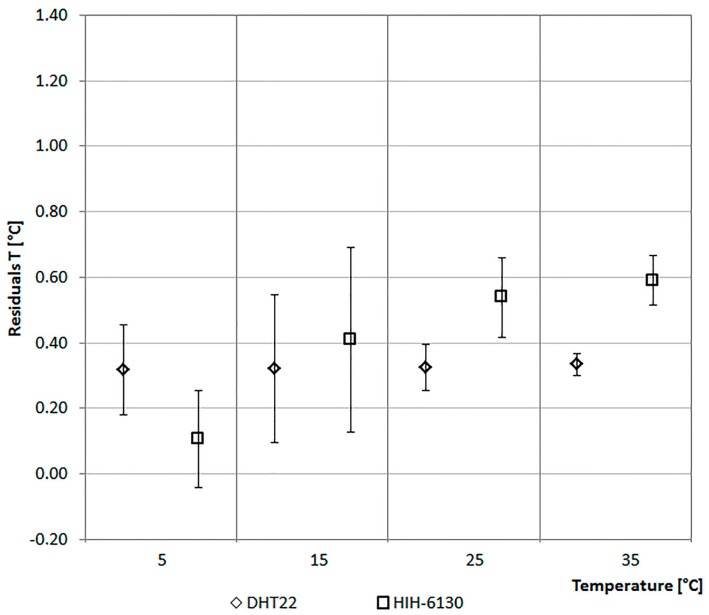
Air temperature data comparison.

**Figure 7:**
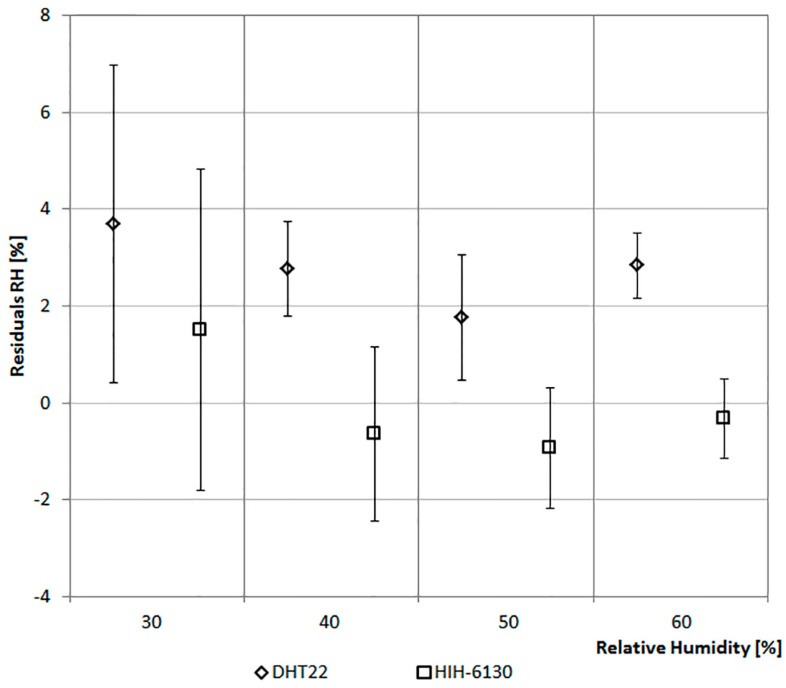
Relative humidity data comparison.

The values of the sensors are compared with that of a climate box. It can simulate different profiles of Ta between −40 °C and +180 °C and of RH in the range from 10% to 98%. Figure 6 and Figure 7 report the considered values of Ta and RH. The ordinate of the graph in Figure 6 reports the difference between the Ta of the climate box and that recorded by the sensor. In Figure 7 the ordinate represents the difference between the RH of the climate box and that recorded by the sensor.

As shown in Figure 6, the monitored values of Ta by the two sensors are, in general, lower if compared with those of the climate box. In particular, the values of DHT22 are lower than the set-points of climate box of about 0.35 °C on the average. The maximum standard deviation of 0.23 °C has been recorded at 15 °C. While the HIH-6130 sensor has a more variable trend across the range with a set-point of Ta equal to 5 °C, the mean and standard deviation are lower than 0.2 °C and they differ at higher temperatures (Figure 6).

Figure 7 shows the trends of RH values for the two sensors. In particular, the values of DHT22 are lower than the set-points of climate box. With a set-point of the relative humidity equal to 30%, it detects the maximum average difference slightly below 4% (standard variation of 3.28%). Considering all the four levels on the average, the trends of RH values for the DHT22 are lower than the set-points of the climate box of about 2.5%. While, the values of the HIH-6130 are closer to the set-points of the climate box. 

In this specific case the analysis has allowed ruling out errors of measurement due to failures of the sensors. It has also allowed reconsidering, for direct comparison in a controlled environment, the choice of thermohygrometric sensor adopted. In fact, in the second optimized case, a DHT22 has been considered in place of the HIH-6130 because it has a more stable behavior over a wide range of TA and RH.

### 4.2. Trad

The globe thermometer used for the measurement of radiant temperature is based on a 10 k thermistor (typical range: −40 to +60 °C; accuracy: ±0.2 °C; average response time: <10 s; long-term stability: 0.02 °C/year). It has been inserted inside a black hollow sphere with a diameter of 40 mm. 

As for the previous case, the values recorded by this low cost globe thermometer (LC_G) are compared to those of a professional globe thermometer (P_G) consisting of of a four-wire PT100 sensor with a copper globe painted black with a diameter of 150 mm. The technical characteristics of the considered globe thermometers are reported in Table 3. 

**Figure 8:**
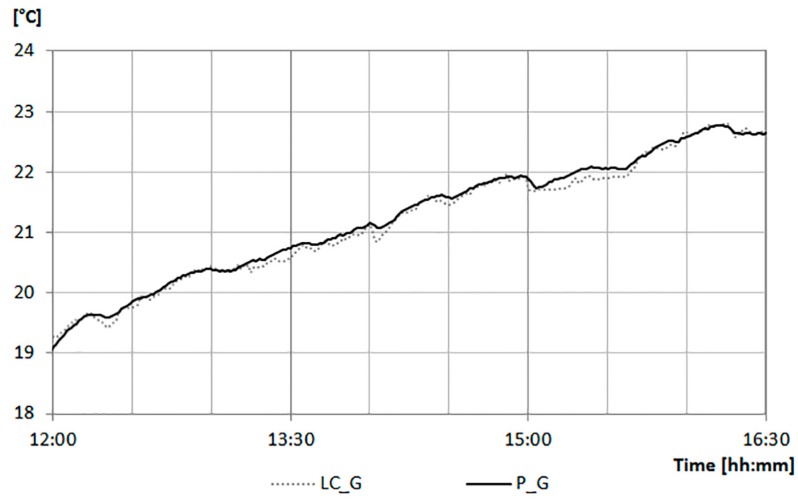
Radiant temperature monitored data: comparison between the low cost globe thermometer (LC_G) and professional globe thermometer (P_G).

The data monitored by both the LC_G and P_G with a period of approximately 4 h are reported in Figure 8.

The difference between the values of LC_G and P_G are less than 2%.

## 5. Optimization and Results

**Figure 9:**
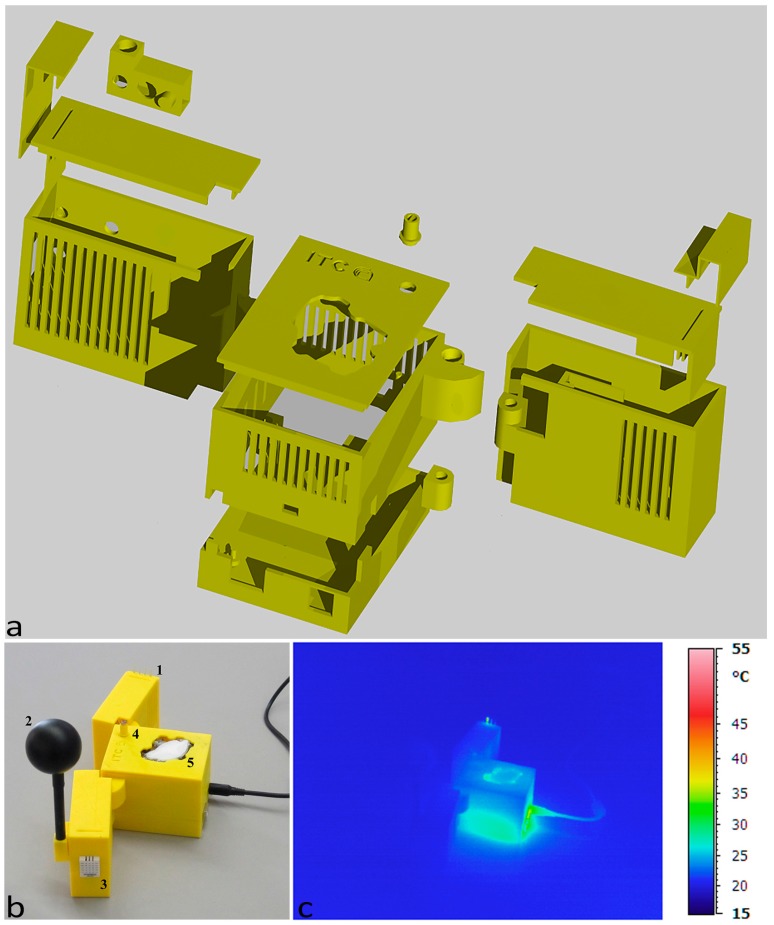
nEMoS case: (**a**) assembling scheme; (**b**) arrangement of sensors; and (**c**) thermal imaging after 24 h.

The calibration phase has allowed ruling out measuring errors due to failures of the sensors. However, the first test of thermographic analysis shows an interference in the measurement of the real temperature due to local overheating. To obviate the above-observed drawbacks, a new case (Figure 9a) was designed and 3D-printed with polylactic acid, a biodegradable thermoplastic derived from renewable resources.

The new case (Figure 9b) of the nEMoS device consists of a central part and two side wings: the end of one wing provides the housing for the anemometer (1), while the globe thermometer (2) and the thermohygrometric sensor (3) are housed in the terminal part of the opposite wing. In this optimized case, the DHT22 sensor is used in place of the HIH-6130. The central part contains the sensors not susceptible to temperature changes, the LDR (4), and the CO_2_ concentration sensor (5). The auxiliary components for the data logging functions are also housed in the central part of the case.

**Figure 10:**
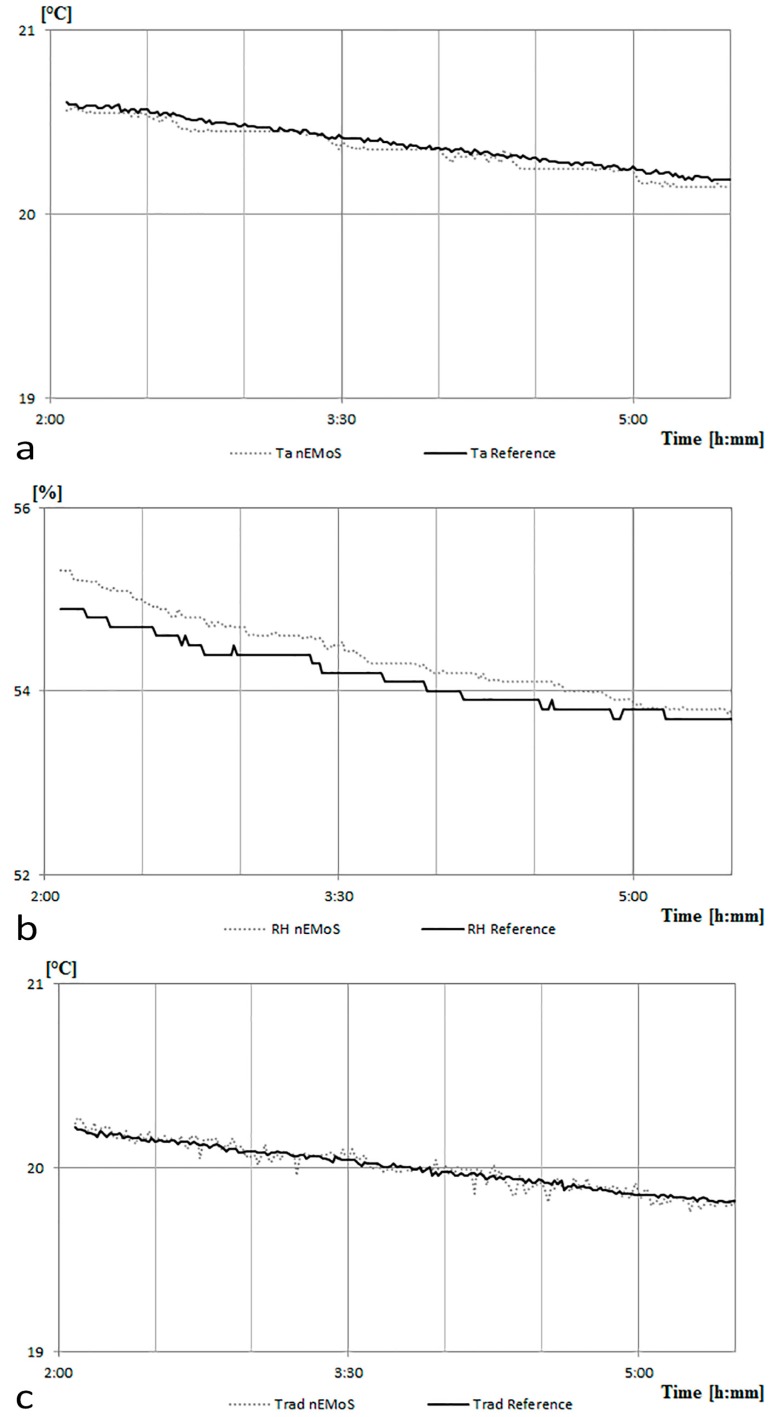
nEMoS Case vs reference sensors: (**a**) air temperature (Ta) data; (**b**) relative humidity (RH) data; and (**c**) radiant temperature (Trad) data.

The thermography performed after one day of operation (Figure 9c) shows no localized heating in the proximity of the globe thermometer and of the air temperature and relative humidity sensor. At this stage, the data of air temperature (Figure 10a), relative humidity (Figure 10b), and radiant temperature (Figure 10c) recorded by the monitoring system compared to those measured by the reference sensors do not show any anomaly.

Even if this instrument is not properly designed to monitor the indoor variables over a long-term, but is designed to assess the indoor environmental data over a shorter time and closest to the positions of end users (i.e., office workstations) [36], a second test was performed after approximately two years (discontinuous use, no exposition to high relative humidity values or to direct sunlight) to verify the long-term stability of this sensor. In this second case, the nEMoS device was located directly in the climate box in order to verify the values recorded by the DHT22 over four different levels of Ta and RH. 

**Figure 11:**
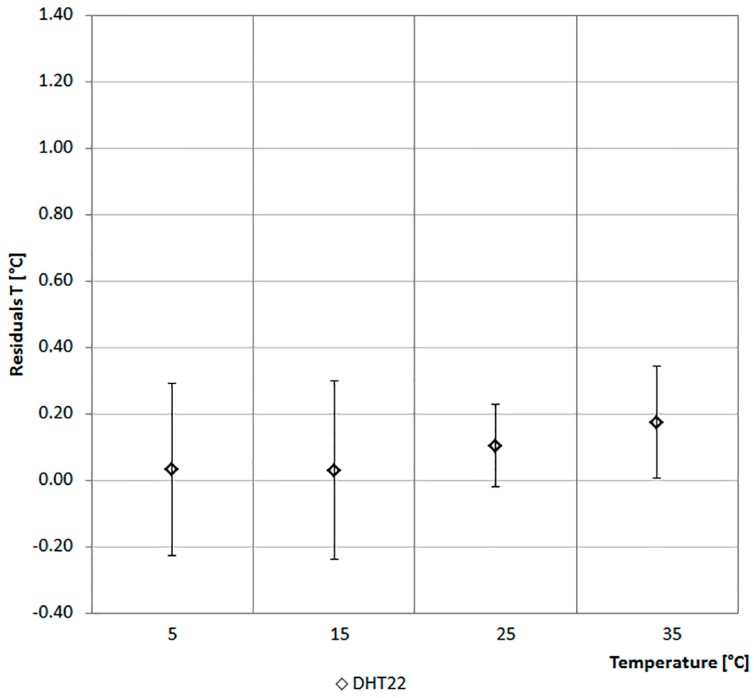
Air temperature DHT22 data after approximately two years.

As shown in Figure 11, the monitored values of Ta by the DHT22 are lower than the set-points of the climate box of about 0.10 °C on average. The maximum standard deviation of 0.27 °C has been recorded at 15 °C. If compared with the values of Figure 6, it is possible to deduce a long-term stability of about ±0.10 ° C/year.

**Figure 12:**
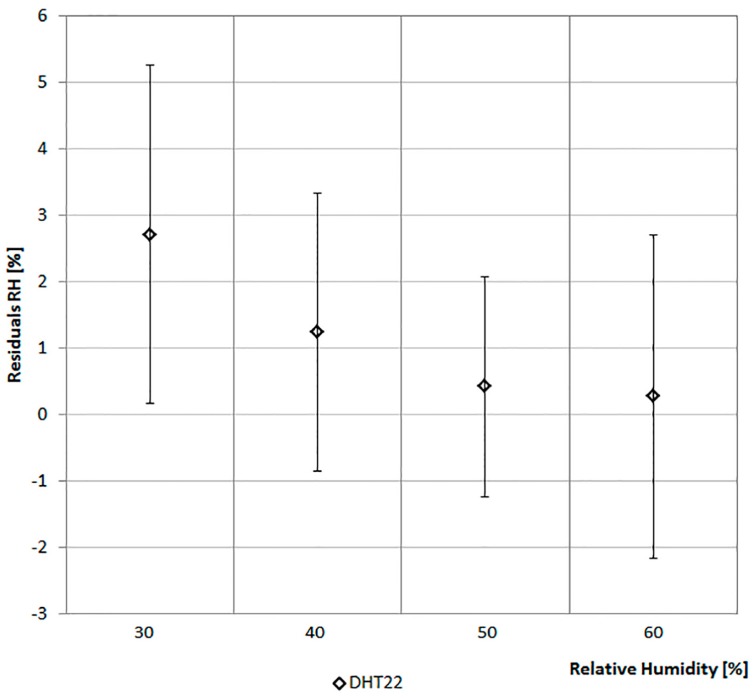
Relative humidity DHT22 data after approximately two years.

Figure 12 shows the trends of RH values for the DHT22 that are lower than the set-points of the climate box of about 1.5% on average. If compared with the average value detected two year before (Figure 7), it is possible to deduce a long-term stability of about ±0.5%/year in line with the value reported in the technical characteristics of this integrated sensor (Table 2). More details about test of DHT22 are available in [37].

## 6. Conclusions

The use of low-cost sensors in replacement of professional ones allows the creation of very small and technologically advanced devices with cloud storage features. However, using low-cost equipment without a preliminary verification of the performance can lead to errors of measurement due to a faulty calibration or to an improper assembly that, as shown, may cause measurement errors due to a combination of sensors with incompatible characteristics. For this reason, infrared technology was used to verify the behavior of the nEMoS device under real operating conditions. In this specific case, through the combined use of additive manufacturing and thermographic techniques, it was possible to detect anomalies in the distribution of temperature and correcting the causes that generated them.

In fact, the thermographic analysis allowed the identification the hottest points, establishing the scope of perturbation due to sensors and circuit elements that heat up the most and, finally, to improve the accuracy of the instrument with a new 3D-printed optimized case.

## Figures and Tables

**Table 1:** Hardware of nEMoS device.

Function/Parameters to Measure	Sensor
Data logging function	Arduino UNO
Web connection	Wi-Fi shield
Bluetooth connection	BlueSMiRF
Air temperature and Relative humidity	HIH6130/DHT22
Radiant temperature	Thermistor within a globe of black color with a diameter of 40 mm
Air velocity	Wind sensor
Illuminance	LDR sensor
CO_2_ concentration	k-30 sensor

**Table 2:** Technical characteristics of the considered thermohygrometric sensors.

Technical Data	HIH-6130	DHT22
**Power supply**	2.3–5.5 V	3.3–6 V
**Typical range**	RH: 10–90%;	RH: 0–100%;
	Ta: −40 to +85 °C	Ta: −40 to +80 °C
**Accuracy**	RH: ±4%;	RH: ±2%;
	Ta: <±0.5 °C	Ta: <±0.5 °C
**Resolution**	RH: 0.04%;	RH: 0.1%;
	Ta: 0.025 °C	Ta: 0.1 °C
**Long-term stability**	-	RH: ±0. 5%/year
**Response time**	Average: 5 s	Average: 2 s
**Dimensions**	18 × 16 × 4 mm (module)	14 × 18 × 4 mm (module)
**Cost**	~30€	~10€

**Table 3:** Technical characteristics of the considered globe thermometers.

Technical Data	LC_G	P_G
**Power supply**	3.3–5 V	10–30 V
**Typical range**	−40 to +60 Celsius	−40 to +60 Celsius
**Accuracy**	±0.2 Celsius	±0.2 Celsius
**Resolution**	-	0.01 Celsius
**Long-term stability**	±0.02 Celsius/year	-
**Response time**	<10 s	<10 s
**Dimensions**	40 mm (ø)	150 mm (ø)
